# Extra Supplement—The Nursing Mirror

**Published:** 1889-05-25

**Authors:** 


*The Hospital,*
MAY 25, 1889.

[*Extra Supplement.*

### The Nursing Mirror.

All Contributions for this Supplement should be addressed "The Nursing Mirror," care of the Editor, 139-140, Salisbury Court, Fleet Street, London, E.C.

### En Passant.

EXAMINATION QUESTIONS.—Three correspondents have written proposing we should give the answers to the examination questions published occasionally in our pages. There are two objections to this. For one thing, the answers would take too much space, and for another thing, we do not know how to secure corrected answers. But if any of our friends who kindly send us specimens of questions will send us answers as well we will be glad sometimes to print them. With regard to a few terms our correspondents have not understood, cluneluvium means a hip-bath, maxilla a jaw, and trocar a stiletto used for tapping. We hope to give some questions set to St. Bartholomew nurses shortly.

SHORT ITEMS.—Miss Mountford, matron, has been elected a life governor of Grimsby District Hospital in acknowledgment of her services in establishing a nursing home, and managing a fancy fair in aid of the new wing.—The full address of the nursing institution at Nice, whose superintendent, Miss Woodcock, wrote to us the week before last, is Hollond Institution, Villa Estradié, Montée de Cimiez, Nice, France.—Dr. Victor Augagner, surgeon at Lyons, has written some articles on nursing, which show the state of this calling in his district. The system of nursing by religious sisters and brethren is dying out, and *infirmiers* are often labouring men, who take a job at the hospital when other work fails them.—The bust of Surgeon-General Gordon at the Academy is the work of Miss C. H. Graham, M.D.—At a meeting of the Dundee Police Commission a minute was submitted from the Sanitary Committee recommending that a matron for King's Cross Hospital be advertised for, her salary to be at the rate of £70 per annum, with board and lodging.

ROYALTY AND NURSING.—The Prince and Princess of Wales always show wise discretion in identifying themselves with different movements, and this is shown with regard to nursing in "Prince, Princess, and People." (Longmans). The Royal family has produced excellent nurses—witness, Princess Alice, Princess Helena, and Princess Christian—and this is doubtless one reason why the subject has received so much notice from them. The Princess of Wales opened the wing of the Deaconesses Institution at Tottenham in 1887, and the Prince and Princess opened the Nurses' Home attached to the London Hospital in the Jubilee year. These homes for nurses have worked a great revolution in the comfort and health of a hard-working band of women, and the Princess was quick to note the importance of the movement. With the work and career of Florence Nightingale the Royal family has always evinced sympathy, and the Queen herself laid the foundation-stone of St. Thomas's Hospital, where the Nightingale school commenced its training. The Prince and Princess of Wales also opened the Marylebone Infirmary in 1881, thus showing their sympathy with the sick paupers who are there nursed by skilled trained women. This infirmary has also an excellent training school for nurses superintended by Miss Vincent. That same year the Prince of Wales was present in the Chapter House at the Abbey, when it was decided to establish a Nursing Institution as a memorial to Lady Augusta Stanley. This was associated with Westminster Hospital, and is now one of our best training schools. It is impossible to state all the incidents of interest which connect Royalty with nursing, but the few enumerated above will prove that it is a subject never forgotten or overlooked.

WIGAN INFIRMARY.—We cull the following items of nursing news from the annual report of the Wigan Infirmary just issued: "The series of lectures by the honorary medical staff to ladies, nurses, etc., has been of an instructive, interesting, and thoroughly practical character, and is becoming one of the features of the institution. An extension of the nursing department was called for in no uncertain voice, and this has happily been accomplished, the six additional dormitories in the Gidlow wing having been completed and are now being furnished. Your board have dealt in such a manner as they think will be of a lasting character with the question of overwork amongst nurses, which has now become a national subject, and at this moment is engrossing the attention of the leading hospital authorities; certainly no more important theme challenges their attention. To bring about the requisite changes, an addition to the nursing staff has been sanctioned. Carefully devised regulations have been adopted, allowing sufficient time for daily outdoor exercise. The assistant matron, Mrs. Neilson, resigned some months ago to fill an appointment to which she was elected—viz., as matron of the Dewsbury Infirmary. Miss Bishop was selected from the nursing staff to fill the vacancy thus caused in recognition of the proficiency shown by her in the examinations on nursing, which were conducted by your medical staff. Four nurses have been set aside for private nursing, and this department works well. The subject of district nursing has been under consideration, but the expenses it involves render it impracticable at present."

NURSES AT BELFAST.—The Society for Providing Nurses for the Sick Poor of Belfast lately held their annual meeting, which was largely attended. The report of the superintendent said: "The twelve months just passed have brought heavy and unintermitting work to both nurses and superintendents. We have attended 897 sick persons, and 31,161 visits have been paid by the nurses. When we mention that amongst these 35 were cases of cancer, 180 ulcers or other wounds, 35 inflammation, and 29 accident cases, the amount of daily nursing required will be readily appreciated; 175 were consumption patients, mostly in the last stage, requiring actual nursing; 200 of our sick people recovered and 192 died; 374 cases were sent to us by medical men. That we have been enabled to increase the number of sick people under our care is owing to the great additional help afforded by the special nurse, who is able to take patients in any district where the work has become too heavy; 1,169 medical and sick-room comforts were lent to our patients." The Mayor moved the adoption of the report, and Dr. Lindsay, in supporting the motion, said that the society was doing a noble work, which could not be done by hospitals. There were no fewer than 800 deaths from consumption in the city each year, and the sufferings of the poor patients were in a great measure alleviated through the efforts of the nurses, who were doing a most necessary work. A great deal of the success of the society was due to the excellent management, and the promptness and attention bestowed upon the cases brought under its notice. There was nothing in the nature of friction between the medical profession and the society, which enjoyed the sympathy of the members of the profession to which he belonged. He had had many opportunities of observing the good which the society was doing, and he could testify to the great benefits it afforded a very deserving class. Financially, the society has had a very successful year.

### The British Nurses' Association and the Press.

The following extraordinary communication was sent by hand to our office at the beginning of the week :

"3, Old Sergeant's Inn, Chancery-lane, E.C.,
"May 20, 1889.

"BRITISH NURSES' ASSOCIATION.

"Sir,—My attention has been called to an article at page xxv of *The Nursing Supplement* to The Hospital of 18th May, reflecting upon the above association. As my instructions are to take immediate proceedings for libel whenever it may be necessary to do so, on behalf of the association, I shall be obliged by your furnishing me with the name and address of 'A Hospital Certificated Nurse' forthwith, that I may lay the matter before the excutive committee of the association and advise them thereon.

"Yours obediently,
"F. S. Randolph.

"T. Burdett, Esq."

Are we to understand from this letter that Mr. Randolph has instructions to examine every newspaper that is published in this country, with a view to taking immediate proceedings for libel, should any editor, in the discharge of his public duty, find it necessary to admit criticism of the methods and working of the British Nurses' Association ?

If so, who is to pay the cost of such legal proceedings against the journalistic world at large, as British editors are sure to continue that course of honest and fair criticism which has made the newspaper press of this country so powerful an agent for good ?

Although we do not hold ourselves responsible for the opinions of our correspondents, still, as we consider the letter complained of to be only a fair criticism on a matter of public interest, we must decline to give up the name of the writer, and must refer Mr. Randolph to our solicitors, Messrs. Slaughter and May, 18, Austin Friars, E.C.

### District Nursing.

### THE METROPOLITAN AND NATIONAL ASSOCIATION.

Once more the Duke of Westminster has shown his interest in nursing by lending his house for the annual meeting of the above association. Unluckily, the Duke himself was not able to preside, but Sir Rutherford Alcock made an able chairman. There was a most distinguished audience present, who murmured approval when it was announced that the association was to have the training of the Queen Victoria Jubilee nurses—an honour the association well deserves, for it stands in the front rank of district nursing institutions. During the year the number of residents in the home had averaged 12¼, while 670 cases had been nursed, and 19,200 visits paid. A branch home had been developed in Chelsea, and the two nurses working at Windsor had been joined by a third. The chairman said he thought that an institution that went on from year to year showing increased activity must in the end be entirely successful. He explained that the nurses of the association, after having been trained in hospitals, were further trained for special work among the poor. He proceeded to speak of the importance of nursing the sick poor in their homes, and, in conclusion, moved that the report be received and adopted. Lord Cobham seconded the motion, which was supported by the Hon. and Rev. E. Carr Glyn, who gave illustrations of the practical work of the institution, and carried. Mr. Caine, M.P., moved a resolution, recognising the good service done by the association, and pledging the meeting to support it in carrying on its work, and in extending its operations. He was glad to know that the association was now out of debt, but funds were urgently needed. Their nurses, he held, were the best in the world. The association had been recognised by the Queen, and there was every reason for maintaining its efficiency. The Rev. E. Venables, rector of Christchurch, Marylebone, seconded the resolution, which was carried. Others were adopted electing the council and thanking the Duke of Westminster for the use of his house.

### Nursing in the Soudan.

*(Concluded from page xxv.)*

### Part VII.—The End of the War.

While crossing the square one morning to breakfast about 8 a.m. we saw a soldier brought in frightfully injured. His horse had thrown him in front of an advancing train, which had gone over him ; his condition was such that he only lived about half an hour. How awful it seemed to us all that the poor fellow should, while in perfect health, die and be buried in one short day. We had many deaths. Fine young fellows losing their lives for such a wretched cause was an unpleasant reflection. A very sad part of it all was writing home to their friends, and sending the pathetic messages with which we were entrusted. "Tell mother I died as a soldier should," said one poor fellow ; while another begged that his mother should not grieve too much. Another—an outspoken, simple Devonshire lad—said, "Mother will miss me. I did all her work for her when at home. Tell her I thought of her to the last." The cemetery was about a mile from the hospital ; and how constantly we saw the funeral party wending their way thither—the officers performing the last sad rite by reading the burial service over the dead ! There was no chaplain to take this duty, or to administer consolation to the dying. As soon as the men were sufficiently convalescent they were sent home in one or other of the ships which were constantly passing.

We had twenty-three enteric cases, officers and men, brought in one night at 12.30. They were on their way home, and when in the Canal were stopped by a barge sinking. Ten days they were subjected to the heat and smells of that unsavoury channel, making some ill, and bringing on a relapse in others. It was difficult to imagine a greater disappointment for them, when having once started for home to be brought back, from such a provoking cause, too. Some of them were very seriously ill. It had been very kindly arranged that the Government steam-launches should be at the service of the hospital authorities three times a week for taking patients out for fresh air in the bay. Accordingly, about 5 p.m., or as soon as it became sufficiently cool, between thirty and forty patients went by train to the harbour, and so on board the launches, accompanied usually by two sisters. This not only did the whole party much good by invigorating and refreshing them, but also gave a great amount of pleasure. Our little service on Sunday seemed much appreciated by the men, and although it was, of course, not compulsory for them to attend, we generally found a good number waiting. The lessons and collects for the day were read, and a short chapter from some suitable book. The choosing of the hymns was left to the men, who seemed to enjoy singing them. The service was held in the verandah outside the different wards that those inside might hear. So the days ran on; June and July passed, August was half through, and our patients had greatly decreased in number—only twenty cases were left—and they were all of a slight character.

Rumours were afloat that the hospital was soon to be closed, for the war had practically ceased. At Suakim we heard they had built a good stone hospital at the point near the open sea: it therefore ceased to be necessary that the men should be sent to Suez when ill. All this pointed to a speedy closing of the Royal Victoria Hospital, and to our departure for home.

In looking back, sad and pleasant memories intermingle, of anxious days and happy ones during our eventful three months. The end of August found us at home again, the proud and happy, though unworthy, possessors of honours conferred upon us both by Her Gracious Majesty the Queen and by the Khedive—the Royal Red Cross, the Egyptian medal with bar, and the bronze star.

### A Nurse's Diary.

### THE FIRST WEEK IN HOSPITAL.

The 1st of Oct., 1883, was a very long day to me. I, having gone through all the form and bother, joined the Birmingham Nursing Institution as a probationer. Dinner at the "Home" (which I did not want), cab to station, another to hospital (on the arrival of train to Wolverhampton), tea, donning the uniform, spending half an hour in the women's surgical ward, supper amid a perfect chaos of voices, prayers in the matron's room—brought one of the longest days in my whole life to a close. The nurses were very nice and kind generally, and yet how funny life is. We all worked, eat, drank, and slept under the same roof, and now (1889) I paid a visit to the same hospital, and only two old faces remain—two charge nurses; all scattered, some filling other posts—two I know in Australia; two or three married. Oct. 2. My first real day of being initiated as a probationer was very trying, my head aching all day. Thankfully I welcomed 12 o'clock, the dinner time. After dinner, in company with a fellow probationer, I went to the dispensary to leave the basket for the patients' medicine; after that I was off duty till four; tea, then ward work again till nine; supper and prayers in the matron's room. Oct. 3.—Pouring all day with rain; visiting afternoon; the friends of the patients from two till four flocked in, much to the wardmaids' chagrin, as all the corridors and wards being polished every morning with beeswax, the wet and mud plays sad havoc. Oct. 4. The work to-day a little more palatable. Went round with the charge nurse Kemp to do the morning dressings; took the basket to the dispensary myself. The dispenser at first sight seemed to have taken on his face a hue from the medicine, pills, and powders. I found the meaning of the text "Judge not," for I afterwards found him genial and kind. After leaving the basket, I returned to the ward and wound a few bandages, in which many convalescent patients assisted. One now took two cups, and went to each person's locker, and took two teaspoonfuls of tea, one in each cup. When all collected, one cup for tea, the other for the patients' breakfast at 5 a.m. Oct. 4. I went off duty till six. Each nurse has an allowance of tea and sugar, and provides her own teapot. From six, when I returned to the ward, till nine, when the night nurse comes on duty, I assisted to take temperatures, give medicines, and do the evening dressings. There are 20 patients in these wards, known as the women's surgical. Oct. 5 Two new cases this morning—abscess in breast and bad knee; all the same routine besides. Oct. 6.—I had a try to bandage a knee. A new charge nurse; the other left. I was on duty all day. Oct. 7.—Nurse Witts in bed poorly; the night nurse stayed on and assisted me to do the dressings. Another probationer sent by the matron from another ward; she and I did the ward work. Nurse Witts still in bed; the assistant house surgeon saw her. The matron paid her usual morning and evening visits. Oct. 8.—My first Sunday in a hospital. Up at six, as usual; on duty at seven. At 10 a.m. the other pro. stayed; I went off duty till 12.30. Charge nurse still in bed. At two I sat on a bench top of stairs, and took the checks from the visitors. At three a bell resounded through the whole building as a warning, the hour of visiting was passed, and the wards resumed their ordinary quiet. The matron, who was very methodical and particular, paid a visit, requesting the friends not to sit on the patient's beds. There is a nice little chapel on the bottom corridor; service always, I learn, in the afternoon. All the convalescent patients, even a few carried, the matron, house doctors, and many of the nurses who can be spared attend. I went this afternoon. It was certainly unique; nurses in uniform, and no one in outdoor dress. Service lasted an hour. Oct. 8.—The house surgeon opened the abscess of the patient who came in on the 5th; also asperated a housemaid's knee. To-day I find I can understand a temperature chart. At two I was off duty; went into the town; all the shops shut, and appears a general holiday, everyone ready to welcome to the town General Booth, the Salvation captain, just come from America. The same routine finished the day in hospital, evening dressings, temperatures, and supper. Oct. 9 Three new cases, all bad knees; the woman with the abscess in breast improving; the knee that was asperated also getting better. One of the new cases, a baby six months; I held it while house surgeon turned its foot and bound it on a splint; it cried very little. The charge nurse still unable to get up. A poor woman who has had a tumour removed is now getting convalescent; asked me to give her a little sewing; have given her wool; she is knitting a pair of socks for my little darling. Oct. 10.—Gas for the first time to breakfast in the day-room. Fearful headache; one new case; I helped her to have a bath, and made a fresh bed for her. A girl's knee that has been asperated several times, the house surgeon opened with a lancet; out immediately came a mass, half as large as an egg, of clotted blood. Charge nurse still unable to come on duty. Oct. 11.—Lovely autumn morning, the small garden lawn belonging to the hospital looks pretty in the autumn sun. One new patient, abscess under arm, very painful. Oct. 12.—Matron gone to the opera at Birmingham. Four new patients; one convalescent gone out. Oct. 13.—Only myself to go round with the house surgeon at ten. Such a quantity of bad knees—one girl of 19 has had hers asperated four times—that is, a needle driven in with a sheath, the needle withdrawn, leaves the sheath, through which runs the collected fluid or serum, unless, as in many cases, it is congealed. Mr. Batterham lanced one case, put the leg on a splint, and a compress on knee. I put the outside bandage on; felt rather nervous. Like everything else, one must have a lot of practice to become an adept even in bandaging. Comparing that first bandage I ever put on and one I put on a few weeks ago for a friend, there is a great difference. My friend told me he had had his leg broken and how painful it still was. At my suggestion his wife got some new flannel (two yards long), and I put on a bandage. After some weeks I met him (I'd forgotten it), and he told me he felt no weakness from the day I put it on, and as I put it, so it still was; walking, working, and sleeping, he was afraid to take it off till he saw me; would I put on a clean one? My first bandage in 1883, though the patient was in bed, slipped all loose by dinner-time. When I came on duty at four, assisted a patient down stairs, who has had a cancer removed for the second time; she is very weak, but is anxious to get home to her young family. Poor woman, hers is a hard and thorny cross. Charge nurse better, night nurse as usual at nine. So closed my first week at hospital work.

### The Nurse's Bookshelf.

"The Hospital Annual," published at our office, price 2s. 6d., should find it way on to every matron's bookshelf at least, and will, we are sure, be very useful to nurses wishing particulars of training schools or institutions. The list of convalescent homes can also be referred to with advantage by nurses or sisters who have difficulties about discharged patients. The tables of comparative expenditure will help many a matron or hospital housekeeper to see where she can best retrench, and which of her bills are truly moderate. As a guide to all hospital, medical, and asylum matters throughout Great Britain we hope this book will be found trustworthy and exhaustive.

### Everybody's Opinion.

[*Correspondence on all subjects is invited, but we cannot in any way be responsible for the opinions expressed by our correspondents. No communications can be entertained if the name and address of the correspondent is not given, or unless one side of the paper only be written on.*]

### THE AGE OF NURSES.

Nurse M. J. writes: As all advertisements are for nurses aged 25 to 35, I often think what must become of all the nurses over that age. Surely no one thinks a nurse done up after that time of life. I am 44, and, after 12 years' general nursing in hospital and private nursing, am still strong and healthy, and trust, with God's help, to be able to nurse for 12 years more. But, then, I am not one of the ill-fed grumblers. I trust hospitals will begin to take nurses of 40, as I know a great many of that age who would be glad to get a good place.

### MAJOR MEARS'S SCHEME.

A. E. S. writes: One knows by long experience how hard it is to induce the public to think that rest and change over and above the yearly holiday are absolutely necessary for hospital nurses; and Major Mears will, like others, grow sad on finding so little interest excited by his scheme. The number of matrons is small who can count much money given for the use of nurses; how few concert tickets, flower show tickets are ever given for the use of hospital workers. Surely there must be many days in a society lady's year when she is not using her carriage and could well spare it for an hour or two? If 50 ladies would band together in every town and send three or four nurses for a drive once a year, what health and pleasure it would give, and how little trouble or sacrifice would it cost. Again, an hour or two on sea or river is only a matter of two shillings, but unless nurses join for a sail, they never get one. Vegetables and fruit given but once a year would confer a great benefit, and not rob either gardener or cook of their lawful (?) perquisites. These remarks have in other words, no doubt, been made before, but with little purpose. A little, very little, thought would add much brightness to the lives of our hardworked and ofttimes tired hospital nurses.

### ALMSHOUSES.

A Torquay Nurse writes: More than once has the subject of "almshouses" for nurses been mentioned in your valuable paper, and, as yet, no such building has been built. Until the "Pension Fund" was started, the future of a worked-out nurse was never thought of, and I fear many of us who are fast coming to the end of our working days will not be able to avail ourselves of that grand offer. Many nurses, through one means or another, have collected articles of furniture or little treasures, and would so delight in even one room to call their own, in which to spend their last days, feeling it was "home," and if there was no rent to pay the delight and comfort would be doubled. If romantic people would cease to gush over the delights of hospital work, and be more real, do something for the nurse when she can no longer work, it would give a stronger belief in the interest they profess to take. I do not believe any nurse, however devoted to her profession, ever saw much that was romantic in washing dirty patients, putting drunken men and women to bed, not to mention a dozen other things which come in the ward work many times in a day, but having chosen nursing as her work, for some reason or other, she does it without a thought as to the pleasantness of it. But to many the day is fast drawing near when we shall be utterly unable for our work, and then, being poor when we started, and not with all our saving (which can only be done by denying ourselves every little pleasure) much richer in the end, what is to become of us? A rent-free room would indeed be a blessing, and our little savings would, in all probability, keep us in "tea and bread," and it is not much more many of us could expect. It is hard that nurses should, during working years, be expected to be "double distilled angels," and then, for all our committees or the general public care, be left to end our days in the workhouse or some miserable room. Those of us who have worked to the best of our ability have, surely, as much right to expect consideration as any other body of public servants, and there are few companies who do not provide for their servants, except hospital committees.

### Lectures.

At Toynbee Hall, Mr. W. Pye, F.R.C.S, is holding a Laboratory Class on "The Histology of the Elementary Tissues," on Fridays at eight. Prof. J. B. Lewis is giving a course of ten lectures on "Practical Chemistry" on Mondays. Mr. E. A. Parkyn is delivering a course of lectures at Gresham College on Mondays, at 6.30, on the "Physiology of Plants."

### Examination Questions.

(The Editor will be glad of specimens of similar questions.)

### JUNIOR MEDICAL.

(1) What is the structure of the human skin? Why does blood flow from it when a leech is put on? How much blood does a leech draw?

(2) What are the parts of the human alimentary canal, and what glands open into it?

(3) What is meant by diet as distinct from food? Write out the diet sheet of this hospital, and say to what groups of food the articles belong.

(4) Where is the spinal cord situated in the body, and what results follow its injury?

(5) How many teeth should a child aged one year have, and how many teeth of what kinds are an adult set composed?

### JUNIOR SURGICAL.

(1) Of what does the blood consist?

(2) Describe the structure of the lungs.

(3) In what portion of the body are the Peritoneum, the Innominate Artery, the Sigmoid Flexure, and the Epiglottis?

(4) What is the difference between a simple, a compound, and a comminuted fracture?

(5) What should a nurse prepare for an antiseptic dressing for amputation of breast?

### Vacancies.

The following vacancies are announced:

**Matrons.**

King's Cross, Dundee, £70.

Beckenham Cottage Hospital, £40.

Brecknock Infirmary, £20.

**Lady Superintendent.**

Hull Royal Infirmary, £70.

**Nurses.**

Grimsby Hospital, £25.
Benges Nurses' Home, £30.
Ramsgate Fever Hospital, £25.
Carmarthen Infirmary, £23.
Cunningham Poorhouse, Irvine, £25.
Barony Parochial Hospital (night), £24.
Eastbourne Nursing Institution.
Providence Nurses' Home, Nottingham.
Nurses' Home, Harrogate.
Brompton Consumption Hospital, £25.
West London Hospital, £24.
Darenth Asylum, £30.
Albert Hospital, Devonport, £25.
Jenny Lind Infirmary, £22.
Hull Nursing Institution, £25.
Kent Nursing Institution.
Workhouse Infirmary Nursing Association.
Salford District Nurses' Home (several), £25 to £28.
Stockport Infirmary (two), £24.
Burton-on-Trent Nursing Institution, £25 to £28.
Kingston Nurses' Home, Hull.
Glamorgan and Monmouthshire Infirmary.
Royal Surrey County Hospital (assistant), £16 to £18.
Bedford Nurses' Institute.
Stratford-on-Avon Hospital (several) £20.

**District Nurses.**

Nursing Society, Ballymena, £30.
Peterborough Association, £30.
Hersham, Walton-on-Thames, £25.
Queen Victoria Institute, Edinburgh.
Red House, Bicester.

**Probationers.**

Lancaster Infirmary, £13.
Burton-on-Trent Nursing Institution.
Stoke-on-Trent Nurses' Home.
Bristol Children's Hospital.
Grimsby Hospital.
Macclesfield Infirmary (several).

### Notes and Queries.

**Queries.**

(16) *Microscope Class.*—Can any of your readers direct me to a thoroughly good class on the use of the microscope? *W.*

(17) *Convalescents from Fever.*—I have a patient just recovering from scarlet fever, where can she be sent for change? *Sister Eve.*

(18) *Massage.*—What is the best book on massage? *Leamington.*

(19) *Nursing in Australia.*—To whom should I apply to secure a post as nurse in Australia? *S.A.B.*

(20) *For the Lepers.*—I see one nurse has volunteered for the service of the lepers. Are nurses required, and under what conditions would they be sent? *M. E. S.*

**Answers.**

(14) *Homes of Rest.*—Apply to Church House, Beaconsfield. *Sister.*

(17) *Convalescents from Fever.*—Try House of Recovery, Carlisle; Convalescent Home, York-road, Leeds; or Mary Wardell Home, Brockley Hill, Stanmore.

(20) *For the Lepers.*—The statement was made by the Secretary of the Church Army, the Rev. W. Carlile. Apply to him, at 130, Edgware-road, for information.

